# Ivermectin and doxycycline treatments against Onchocerciasis: Adaptations and impact among semi-nomadic population in Massangam Health District, Cameroon

**DOI:** 10.1371/journal.pntd.0011463

**Published:** 2023-07-12

**Authors:** Rogers Nditanchou, Ruth Dixon, Kareen Atekem, Benjamin Biholong, Aude Wilhelm, Richard Selby, Joseph Oye, Joseph Kamgno, Daniel Boakye, Elena Schmidt, Laura Senyonjo

**Affiliations:** 1 Sightsavers, Cameroon Country Office, Yaoundé, Cameroon; 2 Sightsavers, Haywards Heath Office, Haywards Heath, United Kingdom; 3 National Programme for the Fight against Onchocerciasis and Lymphatic Filariasis, Ministry of Public Health, Yaoundé, Cameroon; 4 Filariasis and other Neglected Tropical Diseases Research Center, Yaoundé, Cameroon; 5 Faculty of Medicine and Biomedical Sciences, University of Yaoundé I, Yaoundé, Cameroon; 6 Parasitology Department, Noguchi Memorial Institute for Medical Research, University of Ghana, Accra, Ghana; Istituto Superiore di Sanità, ITALY

## Abstract

We trialed strategies to reach semi-nomadic population with interventions targeting onchocerciasis including a combination of community knowledge and Geographical Information System (GIS) technology; nomad-specific sensitization; and mobile outreach. The interventions included ivermectin (ivm) mass drug administration (MDA) and treating infected individuals (found upon skin snip microscopy test) with doxycycline for 35 days. Microscopy-negative snips were further tested by Polymerase Chain Reaction (PCR). After 8 months, individuals immigrating or emigrating constituted 47% of the initial population; 59% of individuals not born in the area have immigrated during the last five years; 28% (age>9) reportedly never taken ivm; 72% (compared to 51% previously) of eligible population (age ≥ 5 years) took ivm; and 47% (age > 8, not pregnant, not breastfeeding, not severely ill,) participated in the test. A high prevalence of onchocerciasis,15.1%, was found upon microscopy & PCR test; 9/10 tested by skin snip microscopy and PCR at follow-up were all negative. Microfilaria prevalence and intensity upon skin snip microscopy reduced significantly from baseline following the intervention (8.9% to 4.1%, p = 0.032; 0.18 to 0.16, p = 0.013, respectively). The strategies considerably increased reach to nomadic camps. Treating with doxycycline in combination with ivm is feasible and has led to a significant reduction in infection level within one year among the semi-nomads. Being potentially curative in one intervention round, this combination should be considered for population group faced with challenges of achieving adequate coverage and adhesion to ivm MDA over prolonged period (>10 years).

## Author summary

Community knowledge, satellite imagery, semi-nomadic sensitization and outreach strategies were employed to deliver ivermectin and doxycycline treatments against onchocerciasis in semi-nomadic population. Doxycycline was offered only when skin snip examination revealed infection. We found that about 50% of the population has either left or joined the community within 8 months; 59% of people not born in the area arrived during the last 5 years; 28% of those older than 9 years reported never taking ivermectin; 72% (compared to 51% previously) of eligible population (≥5 years) took ivermectin; and 47% participated in skin snip test. A high proportion of participants– 15% were infected. All the infected individuals (20) were treated, and no infection was found in almost all those retested (9/10). This study highlights high rate of onchocerciasis and the challenges of offering continuous treatment for a prolonged period (>10 years) to a mobile population. It further demonstrates that combining ivermectin and doxycycline is preferrable in mobile population as it is curative and can be given only one time.

## Introduction

Onchocerciasis is a chronic parasitic disease caused by a nematode, *Onchocerca volvulus (OV)*. OV is transmitted to humans through the bites of infected black flies of the genus *Simulium*, breeding in fast flowing waterways [1]. Prolonged infection can cause skin lesions, severe itching, and ocular disorders, including visual impairment which can lead to irreversible blindness. Although considerable progress has been made in the control of onchocerciasis, there is still an enormous task ahead to achieve the global target of elimination by 2030 [2]. The main strategy for the control and elimination of onchocerciasis is the interruption of transmission through annual Mass Drug Administration (MDA) using the Community-Directed Treatment with ivermectin (CDTi) strategy [3]. However, in areas with unsatisfactory progress, complementary or alternative treatment strategies (ATS) [4] are required to meet elimination targets.

Studies in Massangam in the West Region of Cameroon during 2016 demonstrated unsatisfactory progress. High prevalence of skin micro-filaria (mf) were found in three focal communities—Makouopsap (37.1%), Mankakoun (36.8%) and Njigouet/Njinja (27.5%) after more than 20 years of consecutive annual CDTi [5]. To accelerate elimination, ATS including test and treat with doxycycline (TTd) were implemented [6,7]. A qualitative assessment of the TTd revealed barriers to reaching semi-nomadic population. These included their small, remote and disperse settlements, high mobility, non-inclusion as Community-Directed Distributors (CDD), inadequate consideration in programme planning, low education level, low awareness of onchocerciasis or MDA, dis-trust, and discrimination by settled community, language, and cultural differences [7].

Nomads are members of traditional groups of hunter-gatherers and pastoralists who do not have a fixed habitation and regularly move locations. When there is a base to which the nomads return seasonally, their lifestyle is termed semi-nomadic [8]. Nomadic and semi-nomadic groups often face inequitable access to health interventions [9–11]. Not sufficiently reaching this group for both TTd and ivermectin (ivm MDA) treatments represents an obvious equity concern and threatens elimination efforts. This paper describes adaptations made to improve the reach among semi-nomadic populations and evaluates success (participation), impact (prevalence and intensity) and lessons learnt as recommendations for programmes, guiding integration of similar subgroups in different settings.

## Methods

### Ethics statement

Ethical approvals were obtained from the Ethics Review and Consultancy Committee (ERCC), Cameroon Bioethics Initiative (CAMBIN) (Approval N° CBI/445/ERCC/CAMBIN) and the National Ethics Committee for Human Research in Cameroon (Approval N° 2020/0/1203/CE/CNERSH/SP). Consent forms were read and explained to participants in the language they best understood, and they were encouraged to ask questions which were answered prior to them signing or putting their fingerprint. In addition, parental consent for any individual aged 17 or under was obtained. COVID-19 prevention measures (masks, washing buckets, soap, and hand sanitizers) were mandatory to ensure adherence with government COVID-19 policies [12].

### Setting

**Fig 1:**
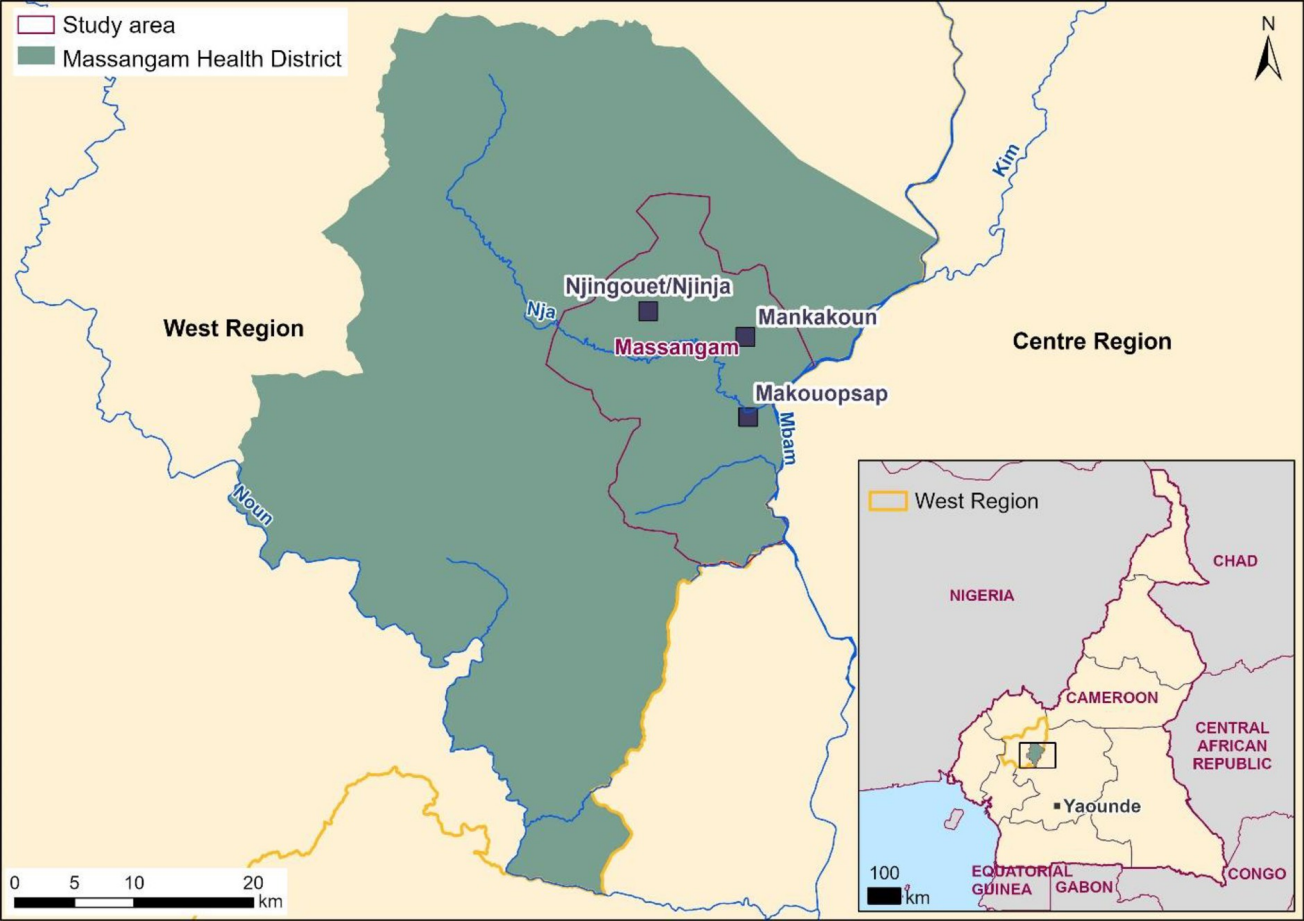
Map of Massangam Health District showing the focal communities within the Massangam Health Area (Sub-District). The blue line indicate rivers. (Sources: Hydrology-HydroSHEDS https://www.hydrosheds.org/; the administrative boundaries–Natural Earth https://www.naturalearthdata.com/downloads/; Community locations were collected during the field activity by using GPS devices. Map was created using ArcGIS software by Esri).

The Massangam HD (Health District) in the West Region of Cameroon is situated in a savannah-forest transition zone. It is characterized by mountainous terrain with altitude between 500 and 1000 m. The Mbam River is predominant in the area. The two main tributaries of the Mbam are the rivers Noun and Nja both of which cross Massangam. These rivers provide year-round breeding sites for *Simulium* blackflies, permitting year-round transmission of *Onchocerca volvulus* [5]. This study was carried out in Massangam health area (subdistrict) (Fig 1) and targeted the semi-nomadic population living in camps around the settled population of the three communities previously determined to have high infection—Makouopsap, Mankakoun and Njijia/Njigouet [5]. Structures within which semi-nomads live are referred to as encampment. Generally, these are groups of huts in which extended semi-nomadic families live. Families are commonly hierarchically organized under a family head. Encampments and the people living in them constitute the semi-nomadic camps.

### Study design

**Fig 2:**
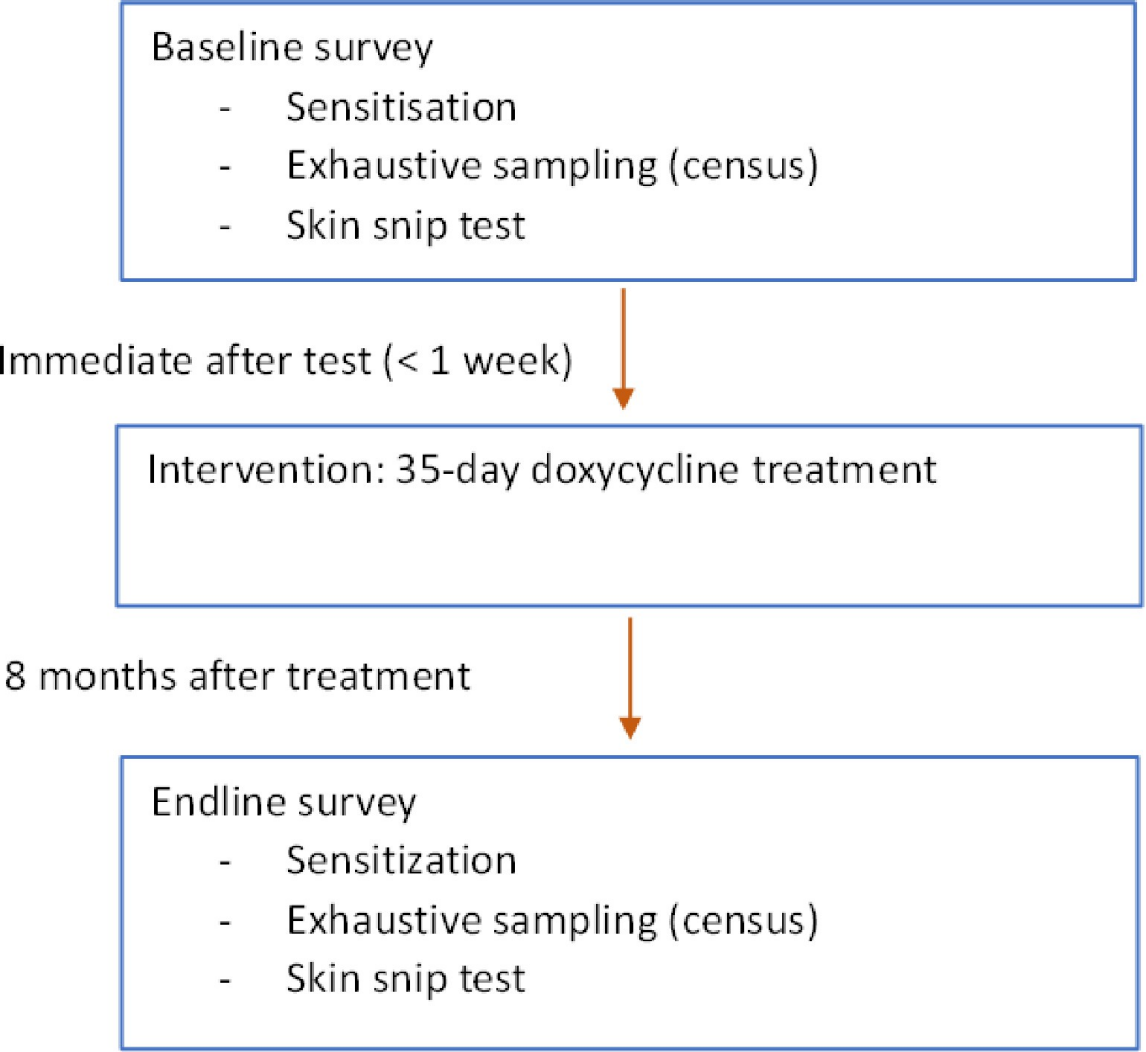
Study design. All ivermectin eligible population (aged 5 years, not pregnant nor breast-feeding children younger than 8 days) including those not tested (skin snip) were offered ivermectin (same day as test).

This study consisted of pre (baseline) and post (endline) intervention surveys. Baseline survey and intervention were conducted from October to December 2019 and endline from August to October 2020. At baseline and endline, sampling was comprehensive including all semi-nomads in every camp and incorporating identification and census of semi-nomads, community engagement, testing of individuals for onchocerciasis using skin snip biopsy (this also served as the baseline survey). Infected individuals were treated with a 35-day course of doxycycline in addition to ivermectin (intervention) (Fig 2).

### Procedure

Prior to skin snip testing, culturally adapted communication materials were developed informed by previous qualitative study [7]. Materials included banners, flyers, and posters. video and audio tapes in four local languages–Pidgin English, French, Bamoun, and Fulfulde were used to reinforce mobilization and sensitization. Reinforcement was done at three levels: district level involving national, regional, and district programme staff, local administrative authorities, and community leaders; community level involving community members; and camp-to-camp sensitization and census in one visit by trained data collectors and community-directed drug distributors (CDDs). We employed a systematic approach to camp identification combining first community knowledge, and secondly GIS (Geographical Information System) technology methods to verify coverage of community identification and close the gap.

### Community knowledge-based identification of camps

Community knowledge methods involved identification of camps by community members (including chiefs and CDDs), listing and locating them on sketch maps. Data collectors were guided by the CDDs (base on their knowledge) to the camp locations. Once in the camps, further efforts were made to identify neighbouring camps that might not have been identified via community listing by asking inhabitants of the camp–a technique termed snowballing. GPS coordinate of community-identified camps visited were collected.

### Geographic Information System-based identification of camps

GIS technology identification involve satellite image identification of potential camps, overlaying with camp location collected during community identification, and then verification of satellite identified potential camp missed (by community methods). Potential camps were identified on satellite image of the area. This identification was based on identifiable characteristic features of encampments previously found [6,7]. Sources of satellite images used included: Hydrology—HydroSHEDS https://www.hydrosheds.org/; (https://www.hydrosheds.org/); and the administrative boundaries—Natural Earth https://www.naturalearthdata.com/downloads/. Locations of community and satellite identified encampments were plotted on the map. Satellite-identified camps not matching community identified location were noted (as community knowledge coverage gap). Missed potential camps were verified with guidance of a bespoke ESRI ArcGIS Explorer live tracking App (https://explorer.arcgis.app/) developed and installed on android smartphone.

To develop the App, first, an office-based exercise was undertaken using ArcGIS software by Esri and satellite imagery to create map of potential encampments missed by the community identification methods. Methodical step-by-step access routes to these remaining encampments were established including transport means (part using the car, the motorbike and by foot). Potential natural barriers like streams or bushes to cross and distance information to complete the roadmap navigation were included. The map was downloaded and installed on smartphone via the ArcGIS online platform for offline use. The App also tracked actual location of the phone on the map using the satellite-based navigation system, GPS (Global Positioning System). With this functionality, field team were able to navigate along the identified route viewing their position until it matched with a (community) missed potential satellite identified encampment. At this point, the team inspect their physical location and surrounding 200m radius searching for camps and if people are living in. As it was the first, the team also had printed back up maps, same as those on the App but with inclusion of latitudes and longitude, and a GPS and compass device in case of technology failure. Snowballing was also done for any camp identified this way. Camps with people living were included in the list of camps for test and treat and ivermectin interventions.

### Skin snip microscopy examination

A screening (skin snip testing) team visited all identified camps after one to three days following camp-to-camp sensitization and census. The team was comprised of a lab technician, an assistant lab technician and a pair of CDDs (one from the settled community and one from the semi-nomadic community). The team overnighted where necessary to allow those absent to participate. Every eligible individual (aged >8 years, not severely ill, not pregnant or breastfeeding) was verbally invited to participate in a screening test. Testing comprised of registration, physical examination, and skin snip (biopsies) collection at the two posterior iliac crests using a 2 mm Holth-type corneoscleral punch. The Biopsies were put into wells of a 96 well-micro-titre plate in which two drops of saline solution had been previously placed and sealed with parafilm to avoid spillage and evaporation. It was then allowed to incubate for 24 hours at room temperature. The incubation medium was examined under a compound microscope (x10) for the presence (infected) or absence of O. volvulus mf [13]. This analysis was performed by the team on field in the camps. The presence (positive) or absence (negative) of onchocercal microfilaria (mf) were noted. If positive all emerged microfilaria (mf) were counted and recorded. All negative snips were transferred into 1.5ml cryotubes onto which an equal volume of isopropanol was added. The tubes were then labelled, packed in Ziplock bags before putting in cool boxes, and transported within 10 days to Filariasis and other Neglected Tropical Diseases Research Center laboratory in Yaoundé where they were stored at -20°C until tested by Polymerase Chain Reaction (PCR) (OV150) [14]. PCR test was delayed due to Covid19 pandemic’s impact on supply chains [15].

All participants provided informed consent before they were screened (tested). During screening, the team continued sensitization explaining to the semi-nomads the purpose of participation. During the baseline survey, basic demographics, residency, travel and ivermectin MDA participation histories were collected.

### Ivermectin and doxycycline treatments

After skin snip, all eligible individuals (aged ≥5 years, not pregnant nor breast-feeding children younger than 8 days) were offered ivermectin (ivm) obtained from the national programme. Ivm was offered to all eligible–including those that refused skin snip procedure, those participating in the test as well as those ineligible for doxycycline treatment. Individuals found mf positive by microscopy were also immediately offered (within less than a week following test) doxycycline 100mg treatment per day for a period of 35 days. Doxycycline treatment was initially provided to participants by CDDs accompanied by the research team, including trained medical professionals. The treatment was observed by the research team for the first two days before handing over to the CDD for weekly monitoring. Light meals were provided before treatment and pictorial treatment diaries were provided to everyone for recording timepoints of self-administration of medication, and how they felt afterwards. Where possible, CDDs made check in phone calls twice a week to monitor adherence and discuss any issues. CDDs made pre-arranged weekly visit to camps, during which they reviewed treatment diaries with each participant. During camp visits, CDDs collected empty and unused doxycycline packets to verify treatment adherence and plan restocking of treatment supplies for the following week. Additional information was collected on reasons for not taking medicines (when required). The CDDs transferred the information from pictorial treatment diaries to a standardised paper treatment register. Researchers collected all treatment registers and diaries upon completion of treatment and later entered the data into an Excel electronic data base.

### Endline survey

Eight months after the end of treatment, all camps were revisited, and all individuals registered at baseline checked and noted if absence, presence, emigrated, or died. Furthermore, new arrivals (immigrants) were recorded in registers. All individuals were invited for follow-up skin snip screening. Consenting and eligible participants had samples collected and examined by microscopy and PCR (of microscopy negative snips). For ethical reasons, ivermectin was offered to everyone consenting and eligible during the test. Those later found positive were additionally treated with doxycycline 100mg for 35 days. These treatment records were excluded from analysis presented in this paper.

### Data analysis

Data was transferred from Microsoft Excel into STATA Statistical Software (StataCorp LLC 4905 Lakeway Drive, College Station, Texas 77845 USA http://www.stata.com), for cleaning and analysis. The data are found in S1 Data.

Population movement (immigration and emigration) were calculated as the proportion of the baseline population who were immigrants or emigrants. Turnover was calculated as the proportion of immigration, emigration and deaths combined, compared to the recorded baseline population. The population age profile was determined by the mean, median, maximum, and minimum ages. The cumulative population was determined as the sum of individuals who were registered at any point (baseline or endline). Ivermectin MDA participation rate was calculated as the percentage of the eligible population (age >5 years) who took medication—number of eligible people who participated divided by total eligible population multiplied by 100.

Participation rate in skin snip test was calculated as the percentage of eligible individuals who took part. Sensitivity of microscopic examination was determined as the ratio that tested positive by microscopy against the total positive by microscopy and PCR combined. Prevalence of OV mf infection at baseline and endline was calculated as the proportion of those testing positive by microscopy divided by those tested multiplied by 100. Prevalence estimate was also calculated for participants that tested positive by either microscopy or PCR.

A multiple logistic regression adjusting for the following variables added a priori: age-group, sex and community, survey points—baseline/endline, and individual participation—single/repeat test categories—was performed to estimate the odds ratio of infection during baseline and endline. Fitness of the model was estimated using LR (Likelihood Ratio) Chi-square test and corresponding p-values (p<0.05 is considered statistically significant). Average mf intensity from microscopy was calculated using the arithmetic mean to obtain mf intensity measures of all skin snipped, inclusive of zero intensity counts. Baseline vs endline average intensities were compared using Mann Whitney U test due to non-normal distribution of intensity data indicated by histograms plot with highly skewed mf intensity distributions at baseline and endline. Fisher Exact Test was used to compare outcome for doxycycline and ivm treatments.

## Results

### Population characteristics

**Fig 3:**
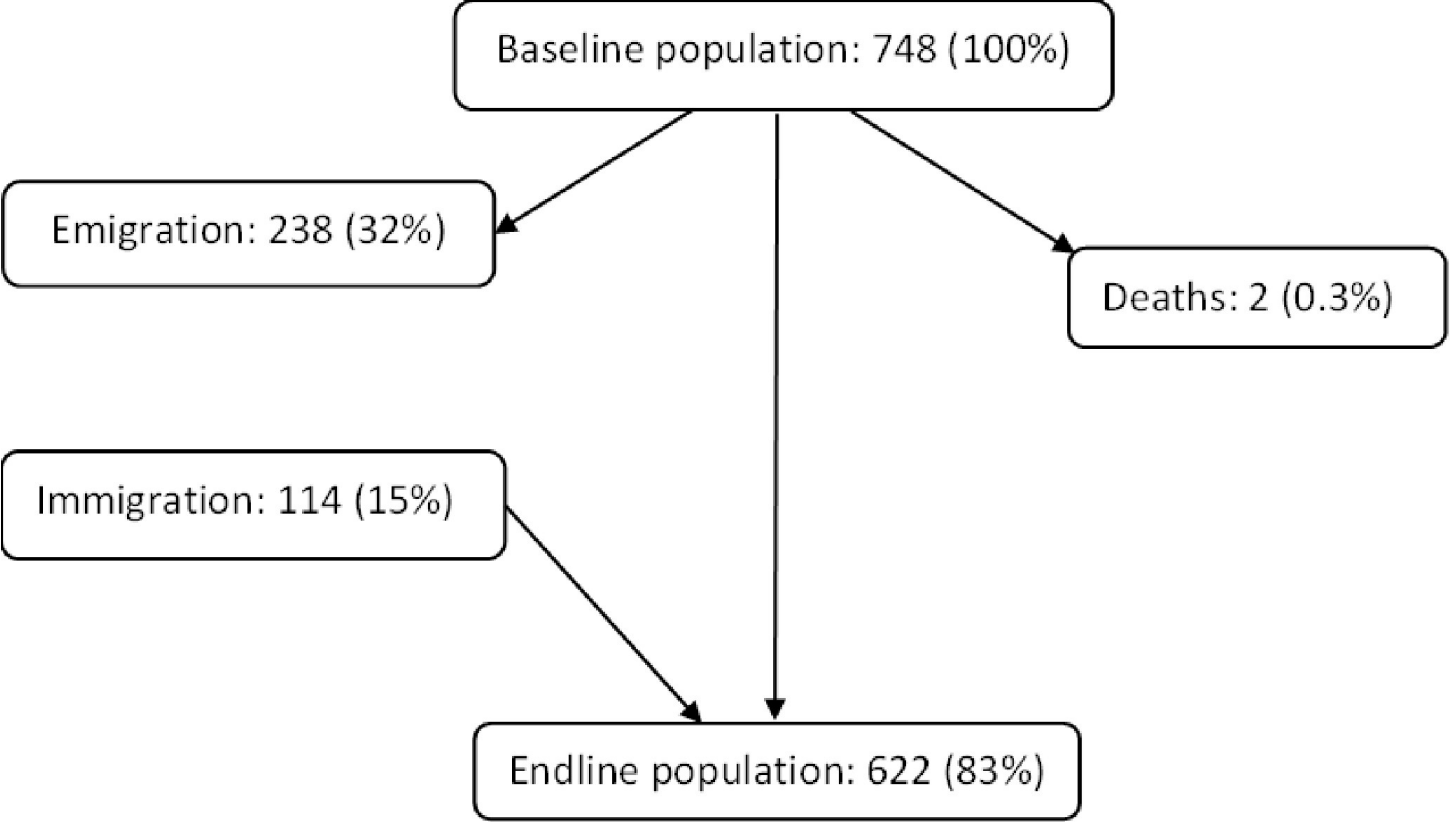
Semi-nomadic population turnover from baseline to endline Percentages are expressed as proportion of the endline population.

Sixty-two semi-nomadic camps were identified, of which 58 were identified through community knowledge and 4 more identified through satellite image. Within camps, baseline and endline census populations varied between 784 and 622 respectively (Fig 3). A population reduction (emigration minus immigration and death) of 17% (n = 126) was noted. The cumulative number of individuals recorded in the area during baseline, endline or both, was 862. Semi-nomadic population was estimated by capture-recapture approach to be 1180 individuals [16]. Population turnover (sum of emigrants, immigrants, and deaths) was 47% (n = 354) of baseline population. Age among the population of 862 ranged from zero to 95 years, mean = 19.5 (+/-17.3) and median = 15 years. Among those not born in the area, 59.0% (CI (Confidence Intervals): 55.3–62.5) (n = 428) have been in the community for 5 years or less.

When asked about participation in ivm MDA for those aged more than 9 years, 28% (CI:24–32) (n = 129) reported they have never participated. Furthermore, only 51% (n = 289) of eligible semi-nomadic population aged ≥5 years reported at baseline that they took ivermectin in the preceding MDA. During the intervention (baseline), 72% (n = 359) of those ≥5 years took ivermectin.

### Participation in the test and treat with doxycycline intervention

**Fig 4:**
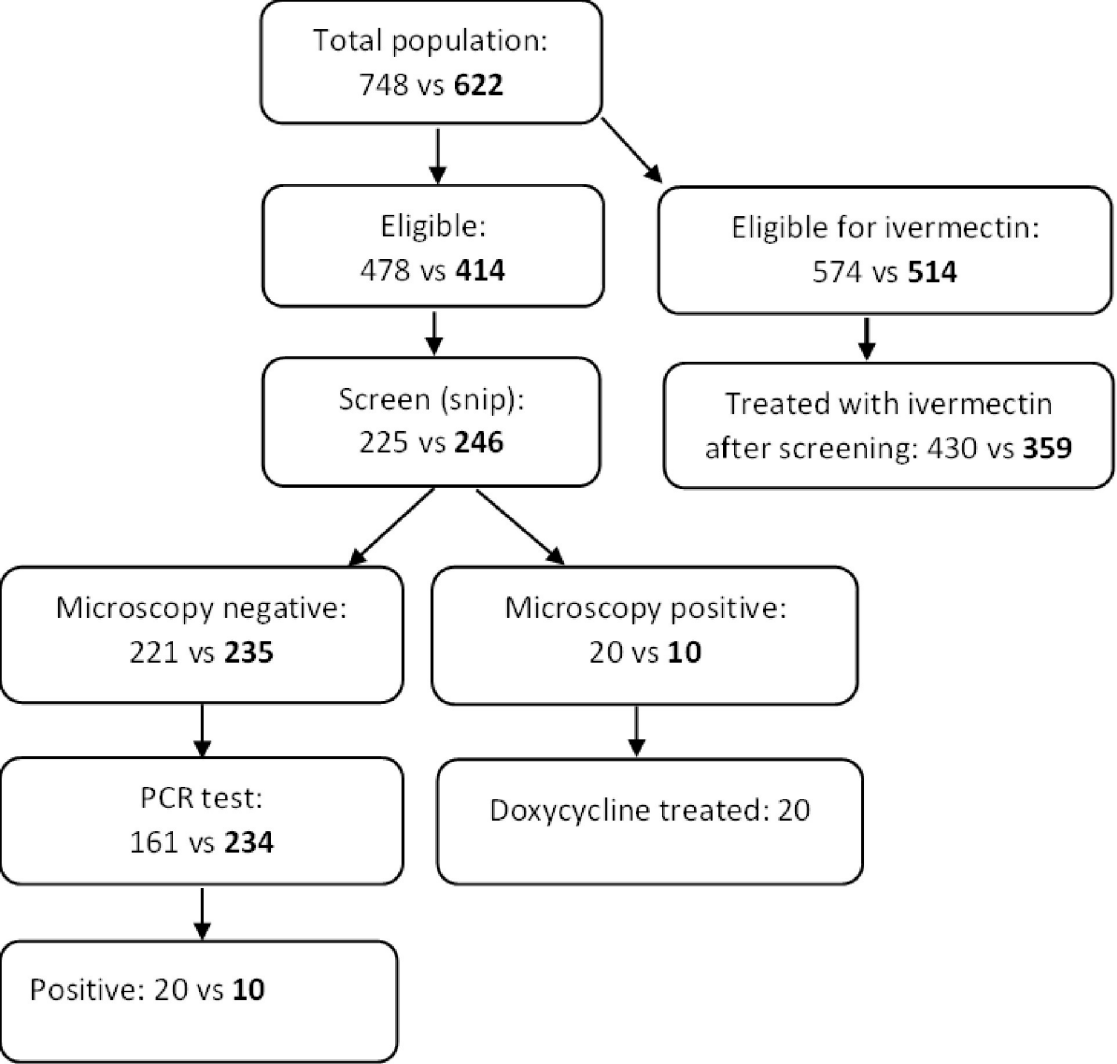
Flowchart showing the total and eligible population, number of participants in skin snip test, number infected and treated at baseline and endline (endline vs baseline) (**endline bold**)

Participation is presented as a flowchart in (Fig 4), clarifying participation from registration, treatment at baseline to endline testing. Test participation among registered, eligible population was 47.1% (n = 225) at baseline, and 59.4% (246) at endline (Fig 4 and Table 1).

**Table 1:** Skin snip test participation at baseline and endline across sex and age categories.

BASELINE					
Categories		# Eligible	# Tested	Percentage tested (CI)	Odd Ratio (CI)*
Overall	Overall	478	225^1^	47.1% (42.6–51.6)	-
Sex	Female	228	104	45.6% (39.2–52.1)	Ref^2^
	Male	250	121	48.4% (42.2–54.6)	1.14 (0.79–1.64; p = 0.49)
Age group^3^	9-15yrs	130	52	40.0% (31.9–48.7)	Ref
	16-25yrs	133	66	49.6% (41.2–58.1)	1.51 (0.92–2.47; p = 0.10)
	26-40yrs	126	66	52.4% (43.7–61.0)	1.64 (0.99–2.72; p = 0.05)
	>40yrs	89	41	46.1% (36.0–56.5)	1.29 (0.75–2.23; p = 0.36)
**ENDLINE** ^**4**^					
**Categories**		**# Eligible**	**# Tested**	**Percentage tested (CI)**	**Odd Ratio (CI)**
Overall	Overall	414	246	59.4% (54.6–64.1)	-
Sex	Female	197	116	58.9% (51.9–65.6)	Ref
	Male	217	130	59.9% (53.2–66.2)	1.04 (0.70–1.55; p = 0.83)
Age group	9-15yrs	124	89	71.8% (63.2–79.0)	Ref
	16-25yrs	117	60	51.3% (42.3–60.2)	0.38 (0.21–0.71; p = 0.002)
	26-40yrs	104	67	64.4% (54.8–73.0)	0.71 (0.38–1.34; p = 0.293)
	>40yrs	69	30	43.5% (32.3–55.4)	0.35 (0.18–0.69; p = 0.002)

1 Out of the 225 tested, 185 (82.2%) received ivermectin.

2 Ref stands for Reference group. It is the group to which the others are compared.

3 Age group odd ratios controlled for length of stay in the community

4. Endline survey 8 months after the end of treatment.

*A multiple logistic regression adjusting for the following variables added a priori: age-group, sex and community, survey points—baseline/endline, and individual participation—single/repeat test categories—was performed to estimate the odds ratio of infection during baseline and endline. Fitness of the model was estimated using LR (Likelihood Ratio) Chi-square test and corresponding p-values (p<0.05 is considered statistically significant).

Of the 34 found positive by microscopy (Table 2) and PCR, 26 (76.5%) received ivermectin. Out of the 8 infected who did not received ivermectin, 3 were detected by microscopy. None of those detected by PCR received doxycycline treated due to delay in obtaining the result.

**Table 2:** Treatment participation among those infected at baseline.

	n	Ivermectin only	Doxycycline only	Doxycycline & Ivermectin	No treated
All	34	9(26.5%)	3 (8.8%)	17 (50.0%)	5 (14.7%)
Microscopy	20	0	3(15.0%)	17 (85.0%)	0
PCR	14	9 (42.9%)	0	0	5 (57.1%)

“All” refers to sum of infected individuals found upon microscopy and Polymerase Chain Reaction (PCR) exam. “Microscopy” refers those found infected by microscopy; and “PCR” to those found infected upon PCR examination of microscopy negative snips. Ivermectin treatments targeted all eligible people (>5 years) irrespective of test or treatment with doxycycline. Meanwhile, doxycycline treatments were offered, in addition, to infected individuals.

Infection was detected by microscopy in 8.9% (n = 20) of participants at baseline. Average infection intensity was 0.18 mf per skin snip. All 20 infected individuals completed a 35-day course of doxycycline treatment (Table 2). There were no reports of adverse effects. Out of the 161 microscopy-negative snips that were cross examined by PCR, 9% (n = 14) were found positive. This gives a microscopy sensitivity rate of 59% (n = 20/34). And an overall detected *OV* mf prevalence of 15.1% (CI: 1.0–20.4) when considering both tests (Table 3).

Ten treated (out 20) individuals at baseline tested at endline and none of them were found positive by microscopy. However, one of these 10 endline microscopy-negative snip was positive upon PCR examination. Out of the nine individuals who were positive upon PCR and took ivm at baseline, five showed up for endline skin snip of which three were negative and one positive upon microscopy and PCR test. Comparing the treatment outcome between doxycycline and ivm groups using Fisher Exact Test (due to small sample size), there was no significant association.

**Table 3:** Comparison of baseline and endline mf prevalence.

	Baseline mf Prevalence	Endline mf prevalence	Prevalence reduction (pearson chi^2^)	Prevalence reduction (adjusted OR**)
Microscopy	8.9% (95% CI: 5.8–13.4)	4.1% (95% CI: 2.2–7.4)	χ2 = 4.58 (p = 0.032)	OR = 0.42 (p = 0.032)
Microscopy or PCR*	15.1% (95% CI: 11.0–20.4)	12.2% (95%CI: 8.7–16.9)	χ2 = 0.85 (p = 0.356)	OR = 0.80 (p = 0.407)
Average intensity	0.18 (0.01, 0.35)	0.16 (0.00, 0.32)	Z = 2.487 (p = 0.013)	

*PCR only on microscopy negative snips

**controlling for age and sex

From 246 participants screened at endline survey, 4.1% (n = 10) were detected as positive by microscopy. This is a significant reduction (p = 0.032) from baseline (Table 3). One individual that was negative upon microscopy at baseline was positive by microscopy at endline. Average infection intensity reduced significantly from 0.18 at baseline to 0.16 (p = 0.013) at endline. Out of 235 individuals tested negative by microscopy at endline and further examined by PCR, 20 were positive- placing sensitivity of microscopy at 33.3% (10/30) and an overall endline prevalence of 12.2% (Table 3). Eighteen (18) (10.7% n = 18/168) were from residents present at baseline but not tested. Eleven individuals infected (14.1%n = 11/78) were detected from new arrivals. When microscopy and PCR results were considered together, the reduction in mf prevalence from baseline to endline was not statistically significant (p = 0.356).

## Discussion

This study revealed a significant population of young mobile semi-nomads, ranging from 862 censused to 1180 when capture-recapture method is applied to estimate the population [16]. The figure of 1180 is conservative as methods for capture (baseline) and recapture (endline) are the same. This leads to high number of overlaps of the population between the two time-points (baseline and endline), thus inflating the denominator with resultant shrinking of the total population estimate. Population turnover was remarkably high within nine months, almost 50% of the baseline population either emigrated, immigrated, or died by endline. This insinuates about half the population are consistently mobile. Accounting for deaths, this mobile population represents either nomadic, or transhumant population [17–19]. The remaining 50% can be regarded as semi-nomadic (have a permanent base that they return to after transhumance) or permanent (historic nomads now settled and do not move seasonally anymore) [20]. As 59% of baseline population reported as having stayed in the community for 5 years or less, migratory, and seasonal movement has been ongoing and is continuing. Even when the nomad settled—no longer moving seasonally, settled local indigenous community still regard them (mostly of Fulani origin) as nomad. Thus, the population can be classified into historic nomads, semi-nomads, nomads, and transhumant nomads. Transhumant refers to mobile semi-nomads or nomads on the move. This distinction of nomadic subcategories, coupled with the significant population size, impacts on onchocerciasis epidemiology and MDA programming and performance.

Nomads are a moving hotspot of infection and transmission and can prolong time to reach elimination or reverse elimination where achieved. An mf prevalence of 15% was found among the semi-nomads at baseline in this study. Of these infected individuals, over 50% were likely to move within a year. This effect is demonstrated among endline participants as 14% of new arrivals were carrying active onchocerciasis infection. Further compounding this problem, the mobile community has a much lower MDA coverage rate. Only 51% of age-eligible nomads in the most recent MDA took part in ivermectin MDA, falling below WHO (World Health Organisation) recommended minimum coverage of 85% among age-eligible population [2,3]. This study also revealed high rate of never treated status as 28% of semi-nomadic population (>9 year) reported never have taken treatment. This is four times higher than that found in settled indigenous population in the same area during 2016 [5,21]. This moving population missed MDA because they are not present during MDA at both ends–where they go to and where they depart from. Also, they can be present during MDA but not included due to their daily movement with livestock and their far, small, remote and disperse settlements. Settled community CDD who often led ivm MDA may not see the need to reach and treat nomads resulting from their perception of them being outsider, troublemakers and destroying their crops. This fuels discrimination, mistrust, and in turn refusal among the nomadic population.

The use of community and satellite camp identification, pairing of CDDs from nomad and settled communities in a buddy-system, adapted sensitization in terms of language and channel, and mobile outreach increase programme reach among the semi-nomads. In addition, delivering ivermectin and test in one outing appeared to improve acceptability and effectiveness of TTd among the nomadic population. More than double the eligible population for testing during previous intervention was reached (n = 212 vs 478). This increased participation is comparable to settled population coverage in previous TTd (47% vs 54%)[6,7]. Additionally, participation in ivermectin MDA delivered alongside TTd led to a coverage increase from 51% of eligible population in previous MDA to 72% in this study. This joint ivermectin and TTd delivery is impactful on both disease morbidity and transmission. This is potentially due to the immediate clearing of mf by ivermectin, and production of new mf gradually reduced from doxycycline impact on the adult worms [22–27]. All but one person retested after treatment was negative suggesting that they may have been cured. However, the evaluation time is short. A longer follow-up is required to ascertain that a sustained and a long-term absence of microfilariae has indeed been achieved. The macrofilaricidal impact of doxycycline makes it superior to moxidectin and ivermectin both of which require repeated rounds over a long period to allow macrofilaria to die. Other macrofilaricidal drugs are being developed [28,29] and once a better one is found, it can be used in place of doxycycline. Doxycycline is part of WHO [4] arsenal of alternative treatments and so we prefer piloting it in programme setting. The one positive individual was only detected by PCR showing that the intensity of infection has reduced. However, it can be argued that mf distribution in the skin is not uniform and false negative upon skin snip sample examination, even with PCR, is likely. The low participation (10/20) of those treated in test at endline survey obscured the impact but we have no reason to believe that impact was lesser among non-participants. An individual having the highest count per snip was among those retested and was not found positive by both PCR and microscopy.

The reduction in average infection intensity and prevalence indicates a reduction in transmission [30]. Microscopic assessment that guided treatment decision at baseline missed at least a third of infected individuals given 33.3% prevalence consider both microscopy and PCR recorded in this study. If these missed individuals were detected and treated, reduction in prevalence and intensity would have been more pronounced. This missed opportunity add to the call for development of a more sensitive field test for detecting active onchocerciasis infection. Delivery of such a test, when combined with a curative intervention such as TTd, is relevant to nomadic and other hard-to-reach populations. In the interim, OV16 test can be used for TTd in treatment naïve individuals.

## Conclusion

This study investigates a large nomadic population, finding a high infection of onchocerciasis. This is a threat to achieving elimination or even reversing its achievement. The strategic adaptions employed in this study have increased effectiveness and acceptability of ivermectin and test and treat with doxycycline. This has driven down infection intensity, and thereby, transmission of onchocerciasis. Reinforced sensitisation, combined GIS and community knowledge identification of nomad camps, and mobile outreach should be considered for inclusion into routine programmatic operations to reach nomadic and similar hard-to-reach populations. Future study should explore further the nomad populations, identifying different nomadic groups such as nomads, semi-nomad, transhumant, historic (settled) nomads to better orientate interventions. Test and treat with doxycycline, in combination with ivermectin treatment should be considered among populations presenting challenges of effective and regular ivermectin treatment participation. Development of a more sensitive and less invasive test for detecting active onchocerciasis infection will result in greater impact of test and treat with doxycycline.

## Supporting information

S1 Table Individual skin snip data. (XLS)
