# Correction: Ivermectin and doxycycline treatments against Onchocerciasis: Adaptations and impact among semi-nomadic population in Massangam Health District, Cameroon

**DOI:** 10.1371/journal.pntd.0011980

**Published:** 2024-02-22

**Authors:** Rogers Nditanchou, Ruth Dixon, Alexandre Chailloux, Kareen Atekem, Benjamin Biholong, Aude Wilhelm, Richard Selby, Joseph Oye, Joseph Kamgno, Daniel Boakye, Elena Schmidt, Laura Senyonjo

Alexandre Chailloux should be included in the author byline instead of the Acknowledgments. Alexandre Chailloux should be listed as the third author, and his affiliation is:

Sightsavers, 36 Perrymount Road, Heywards Heath, RH16 3BW

The contributions of this author are as follows: conception, implementation, and analysis in this article. All the map-related resources and products have been provided/developed by him.

The correct citation is: Nditanchou R, Dixon R, Chailloux, A, Atekem K, Biholong B, Wilhelm A, et al. (2023) Ivermectin and doxycycline treatments against Onchocerciasis: Adaptations and impact among semi-nomadic population in Massangam Health District, Cameroon. PLoS Negl Trop Dis 17(7): e0011463. https://doi.org/10.1371/journal.pntd.0011463
