# Supplementary material for: Ivermectin and doxycycline treatments against Onchocerciasis: Adaptations and impact among semi-nomadic population in Massangam Health District, Cameroon
Source: PLoS Negl Trop Dis. 2023 Jul 12;17(7):e0011463. doi: 10.1371/journal.pntd.0011463 (PMC10365309; doi:10.1371/journal.pntd.0011463)
Supplement: Attachment — Submitted filename: Response letter PNTD-D-23-00095.docx [file pntd.0011463.s002.docx]

 **PNTD-D-23-00095, Test and treat with doxycycline strategy for Onchocerciasis: adaptations and impact among semi-nomadic population in Massangam Health District, Cameroon**.

**Responses to reviewers’ comments**

Dear Editor,

We thank you and the reviewers for your precious time spent to review this manuscript and providing valuable feedbacks and insightful comments. We have carefully considered the comments and address to the best of our ability every one of them. We hope the manuscript after revision meets the standard. The responses to the comments raised are detailed below. They are arranged in a comment-response manner, with line references from the version with track changes. We have include a version with changes highlighted as track and a clean version

 We welcome any further constructive comments.

Sincerely,

*RN*

Rogers NDITANCHOU

**Methods**

-Are the objectives of the study clearly articulated with a clear testable hypothesis stated?

-Is the study design appropriate to address the stated objectives?

-Is the population clearly described and appropriate for the hypothesis being tested?

-Is the sample size sufficient to ensure adequate power to address the hypothesis being tested?

-Were correct statistical analysis used to support conclusions?

-Are there concerns about ethical or regulatory requirements being met?


**Reviewer #1:** The method is well described. The statistical analysis is appropriate.

Response: Thank you.

Could you please clarify by whom the satellite images were provided?

Response: Thank you. Sources of satellite images included: Hydrology — HydroSHEDS <https://www.hydrosheds.org/>; and the administrative boundaries — Natural Earth <https://www.naturalearthdata.com/downloads/>. This has been included in lines 139-141

Could you please give more information regarding the ESRI ArcGIS Explorer live tracking App?

Could you please give more details on how it was used? This is not very clear.

Response. Thank you. More information and how the ESRI ArcGIS Explorer live tracking App was used is included in lines 146 – 161.

Could you please indicate where ivermectin was sourced?

Response: Thank you. Ivermectin was obtained from the national programme. This is included in lines 188-189.

line 212 could you please clarify what statistical test was used to determine the non-normal distribution of intensity data?

Response. Thank you. This was done by histograms plot indicating highly skewed mf intensity distributions at baseline and endline. This is included in lines 246-247.

Please correct the Error reference at line 119

Response. Thank you. This is figure 2. Included now in line 119.

Could you please add in the supplementary material the individual data concerning the skin snips? I believe this is essential information to be included.

Thank you. This has been added – removing identifying information (names and GPS coordinates); lines 221; 507-508.

Reviewer #2: - Lines 129-136, the methodology used to identify enchampments should be further detailed. Was it there a systematic approach? How was conducted?

Response. Thank you. We employed a systematic approached to camp identification that combines community knowledge and GIS (Geographical Information System) technology methods. This has been elaborated in lines 146-161.

- Line 152-154, How were snips preserved and transported? What were the conditions? Where was the test conducted?

Response. Thank you. We have included in lines 180-184.

- Line 156-159, the authors might have missed up ivermectin and doxycycline? Otherwise, I suggest to rephrase that part.

Response: Thank you. We have rephrased; lines 187-191.

- Line 185, authors should include in the discussion how this might have affected their results.

Response: Thank you. We have included this 386-387.

- Line 196-197, what was the denominator (population) considered for that calculation?

Response: Thank you. It is total eligible. This has been included in lines 228-230.

- Lines 205-212, what were the variables considered for the multiple logistic regression?
Response: Thank you. They were age-group, sex, and community, survey point -baseline/endline, test – single/repeat categories; included in lines 238-240.

Reviewer #3: The methods used in this study are well suited to address its key questions. There are no major analyses requested, however (see Results review section) I would suggest adding another table with patient that were present at both beginning and endpoint.
Response: Thank you. This has been added; table 2, lines 285-293.
--------------------

**Results**

-Does the analysis presented match the analysis plan?

-Are the results clearly and completely presented?

-Are the figures (Tables, Images) of sufficient quality for clarity?

Reviewer #1: The statistical analysis is sufficiently described.

Response: Thank you.

It would be useful including few additional details in table 1:
1. Although the end-line survey is described in the method section, it would good to indicate that the end-line survey occurred 8 months after the end of treatment.

Response: Thank you. This has been included; line 283-284.
2. Could you please clarify what is Ref?

Response: Thank you. Ref stands for Reference group. It is the group to which the others are compared; line 282-283.
3. Could you please indicate the percentage of patients who received ivermectin, doxycycline, or no treatment?

Response: Thank you. We have added this in table 2; lines 285-293.

Please correct the Error reference at line 212, 227, and 242.

Response: Thank you. This has been corrected. 262, 276, & 279

At line 207 please use the appropriate symbol for Chi-square and not X2

Response: Thank you. This has been corrected, line 241-242.

Reviewer #2: (No Response)

Reviewer #3: The results are clearly presented and understandable for me. I would however suggest that another table be put together in which only the patents are listed that were already present at study onset and to whom the drug intervention (doxycycline or ivm only) was administered. As the authors write, because of the nomadic lifestyle many patients participated only at the endpoint time, so that this obscures the success of the previous treatment.

Response. Thank you. We have included this in table 2 and lines 285-293.

There are a few minor points that also need attention:
Line 145: sentence incorrect

Response: Thank you. This is corrected, line 173-174.

Line 178: really all patients could be found again after 8 months? In a semi-nomadic population? Does not match with line 226 (this is plausible)

Response: Thank you. We have reworded this; line 209-210

Line 317: check grammar

Response: Thank you. This is corrected; line 361.

Line 330: check spelling: must read “markers”
Response: Thank you. This is now removed in line with reviewer #1 comments below.
--------------------

**Conclusions**


-Are the conclusions supported by the data presented?

-Are the limitations of analysis clearly described?

-Do the authors discuss how these data can be helpful to advance our understanding of the topic under study?

-Is public health relevance addressed?

Reviewer #1: line 323-324 the cited reference (10.1002/14651858.CD011146.pub2) doesn't support '[...]evidence on the effectiveness of doxycycline plus ivermectin compared with ivermectin alone in preventing and treating onchocerciasis [...]'. 'Limited evidence of very low quality from two studies indicates that a six‐week course of doxycycline followed by ivermectin may result in more frequent macrofilaricidal and microfilaricidal activity and sterilization of female adult Onchocerca compared with ivermectin alone; however, effects on vision‐related outcomes are uncertain'.
I would be therefore more circumstantial regarding the parasitological effect of doxycycline, especially because the end-line survey occurs only 8 months after treatment.
If available, it would be very interesting to include information regarding the clinical benefit (itching, presence of microfilaria in the eyes, etc...) or at least mention this point in the discussion.

Response. Thank you. We do not have data of clinical status. We have revised the statement; lines 369-377

lines 325-326 I don't think there is enough evidence to support that patients were cured. A longer follow-up would certainly be required to ascertain that a sustained and a long-term absence of microfilariae has indeed taken place.
Response: Thank you. We have modified; lines 369-375

lines 329-330 As far as I can see reference 10.1371/journal.pntd.0010380 describes a correlation between skin snip and Ov16 ELISA in children (<10). The article describes such correlation in the context of indicators to stop an MDA campaign based on evaluation in children between 5 to 10 years. Furthermore, the cited article describes a variation of anti-Ov16 IgG4 levels with age and the microfilariae load.
In table table 1 this group of patients is not described, what is the evidence that these testes are correlated in adults? And what is the evidence they can be used as endpoints to assess cure? Please clarify this point.

Response: Thank you. We have withdrawn the statement.

Reviewer #2: (No Response)

Reviewer #3: Conclusions are appropriate.

--------------------

**Editorial and Data Presentation Modifications?**


Use this section for editorial suggestions as well as relatively minor modifications of existing data that would enhance clarity. If the only modifications needed are minor and/or editorial, you may wish to recommend “Minor Revision” or “Accept”.

Reviewer #1: (No Response)

Reviewer #2: (No Response)

Reviewer #3: (No Response)

--------------------

**Summary and General Comments**


Use this section to provide overall comments, discuss strengths/weaknesses of the study, novelty, significance, general execution and scholarship. You may also include additional comments for the author, including concerns about dual publication, research ethics, or publication ethics. If requesting major revision, please articulate the new experiments that are needed.

Reviewer #1: I think this work is remarkable. Reaching nomad populations living in remote areas and excluded from the preventive chemotherapy program is certainly a huge challenge.
I am however not entirely convinced by the conclusions and the discussion. Doxycycline is not a first-line treatment for the treatment of onchocerciasis and its parasitological effect is not yet fully proved. Other molecules with an anti-Wolbachia mechanism of action are currently evaluated in clinical trials and there will be more insight regarding the parasitological efficacy in the coming years. It would be therefore important to explain what are the advantages of doxycycline for this population and to justify why in this particular context doxycycline is perceived to be a better option in comparison to ivermectin or moxidectin. Especially, because in this study it is not possible to compare the treatment outcome of patients treated with ivermectin versus doxycycline.

Response: Thank you. Doxycycline is commonly and widely used antibiotics and has been shown to have impact of onchocerciasis macrofilaria. The macrofilaricidal impact of doxycycline makes it superior to moxidectin and ivermectin both of which require repeated rounds over a long period to allow macrofilaria to die. Also, doxycycline has been advised by WHO (<https://apps.who.int/iris/bitstream/handle/10665/343028/WHO-MG-15.20-eng.pdf?sequence=1&isAllowed=y> ) as part of alternative treatment arsenal to consider. Any better macrofilaricidal drug is welcome. We prefer to pilot WHO recommended treatment in a programmatic setting. See lines 369-380.

Reviewer #2: This study is very relevant for onchocerciasis elimination and should be published. The rationale of the study is very well written and easy to follow. There are minor comments that I think that should be included in methodology.

Response: Thank you. We have addressed the comments.

Reviewer #3: This is a very important paper, addressing the problems of adherence to yearly mass drug administration of ivermectin for onchocerciasis. It shows that only around 50% of this population had taken part in MDA and therefore analyzed the success of a curative treatment with doxycycline. The data show that in 9/10 patients who received doxycycline, microfilaridermia was absent at the end of the follow up period (endpoint). The data support the use of macrofilaricidal / long-term sterilizing treatments for mobile/hard to reach populations.

Response: Thank you.

--------------------
Reviewer #1: No

Reviewer #2: No

Reviewer #3: Yes: Prof. Achim Hoerauf, Institute for Med. Microbiology, Bonn University Medical Center, Germany
