# Supplementary material for: Ivermectin and doxycycline treatments against Onchocerciasis: Adaptations and impact among semi-nomadic population in Massangam Health District, Cameroon
Source: PLoS Negl Trop Dis. 2023 Jul 12;17(7):e0011463. doi: 10.1371/journal.pntd.0011463 (PMC10365309; doi:10.1371/journal.pntd.0011463)
Supplement: Attachment — Submitted filename: Response letter PNTD-D-23-000951R1.docx [file pntd.0011463.s003.docx]

**PNTD-D-23-00095R1, Test and treat with doxycycline strategy for Onchocerciasis: adaptations and impact among semi-nomadic population in Massangam Health District, Cameroon**.

**Responses to reviewers’ comments**

Dear Editor,

We thank you and the reviewers for your precious time spent to review this manuscript and providing valuable feedbacks and insightful comments. We have carefully considered the comments and addressed to the best of our ability every one of them. We hope the manuscript after revision meets the standard. The responses to the comments raised are detailed below. They are arranged in a comment-response manner, with line references from the version with track changes. We have included a version with changes highlighted as track and a clean version.

 We welcome any further constructive comments.

Sincerely,

*RN*

Rogers NDITANCHOU

**Reviewer's Responses to Questions**

<b>Key Review Criteria Required for Acceptance?</b></br></br>
As you describe the new analyses required for acceptance, please consider the following:</br></br>

<b>Methods</b></br></br>
-Are the objectives of the study clearly articulated with a clear testable hypothesis stated?</br>
-Is the study design appropriate to address the stated objectives?</br>
-Is the population clearly described and appropriate for the hypothesis being tested?</br>
-Is the sample size sufficient to ensure adequate power to address the hypothesis being tested?</br>
-Were correct statistical analysis used to support conclusions?</br>
-Are there concerns about ethical or regulatory requirements being met?</br></br>

**Reviewer #1:** The authors have nicely implemented suggestions from reviewers

Response. Thank you.
**Reviewer #2:**

- Ethics, all participants were literate? Otherwise, the approach followed should be

detailed here.

Response: Thank you. We have amended; lines 101-104
- Community listing in addition to GIS identification of camps is a very approapiate technique using the resources availables. However, in my opinion, the "Procedure" it is not clearly stated. I suggest to clearly divide the different ideas among the different paragraps. It is quite difficult to follow at the moment.

Response: Thank you. We have adjusted, splitting into subheading; lines 140-182
- Site of analysis or biopsis is not stated.

Response: Thank you. Biopsies were analysed in the field. We have included in line 196-197
- Storage conditions of samples during transportation it is not explained.

Response: Thank you. We have amended: 199-204
- Microscopy technique used is not stated.

Response: Thank you. We have adjusted; lines 192-196.
- Line 248, "Multiple logistic regression", would they mena multiple logistic regressions or amultivariable analysis? This could be clarified. They should also mention if it was an adjusted analysis and the variables they used to adjust for.
Response: Thank you. A multiple logistic Regression adjust for the factors included (see https://www.sciencedirect.com/science/article/abs/pii/0021968181900400). We have adjusted; line 263-266
Reviewer #3: Ok, as in the original version. I noticed methods have become more detailed, probably at the request of other reviewers.

Response: Thank you.
--------------------

<b>Results</b></br></br>
-Does the analysis presented match the analysis plan?</br>
-Are the results clearly and completely presented?</br>
-Are the figures (Tables, Images) of sufficient quality for clarity?</br></br>
**Reviewer #1:** The authors have nicely implemented suggestions from reviewers

Response: Thank you.
**Reviewer #2:** - Table 1, the authors should clarify how the OR was calculated and what variables were used.

Response: Thank you. We have elaborated this in lines 263-272 and again in table 1 legend: lines 300-304.
- Figure 1 could be substitued with a map of proportion of infectes/proportion of tested/treated per region.

Response: Thank you. This figure shows study area. In addition, the study was performed in one health area and analysis as such. We have adjusted figure 1 for clarity

**Reviewer #3:** A new Table 2 has now been added, this is helpful. However, the numbers do not add up: the numbers in the top line must be the summaries of the lines below, e.g. if Ivermectin “all” =9, of those pos. with microscopy = 0, then those pos. in PCR must have again 9 treated with ivermectin, not 6.
Response: Thank you very much. This has been adjusted. Table 2.
Further, it is written that “All 20 infected individuals completed a 35-day course of doxycycline treatment (table 2). “ One would really like to know how many of them appeared at follow up, and whether they were MF pos. or not, in comparison to the 9 PCR-only positives who received IVM only. Even if numbers are small and do not become significant, it would help interpret the data (btw., I have done a Chi-square out of curiosity if one could obtain a significant difference between doxy treatment and ivermectin-only, and indeed even with these small numbers a difference could become significant if most IVM-treated stayed MF pos. while the doxy (±IVM) treated became MF neg.).

In conclusion, the authors need to rectify Table 2 and tell us about the outcome of the patients which they listed in Table 2.

Response: thank you very much. We have expanded on this; lines 321-327
--------------------

<b>Conclusions</b></br></br>
-Are the conclusions supported by the data presented?</br>
-Are the limitations of analysis clearly described?</br>
-Do the authors discuss how these data can be helpful to advance our understanding of the topic under study?</br>
-Is public health relevance addressed?</br></br>

Reviewer #1: The authors have nicely implemented suggestions from reviewers

Response: Thank you.

Reviewer #2: Conclusions are approapriate.
Response: Thank you.

Reviewer #3: Conclusions are fine but need the support as detaled in what I wrote for the Results section.
Response. Thank you. We have adjusted as advised.
--------------------
<b>Editorial and Data Presentation Modifications?</b></br><br/>
Use this section for editorial suggestions as well as relatively minor modifications of existing data that would enhance clarity. If the only modifications needed are minor and/or editorial, you may wish to recommend “Minor Revision” or “Accept”.

Reviewer #1: (No Response)
Response: Thank you.

**Reviewer #2:** There might be a typo error in line 90: The exclusion of this group from both TTd and ivermectin (ivm MDA treatments represents an obvious equity concern and threatens elimination efforts.
Response: Thank you. We have adjusted, lines 90-91.
**Reviewer #3:** (No Response)

--------------------
<b>Summary and General Comments</b></br></br>
Use this section to provide overall comments, discuss strengths/weaknesses of the study, novelty, significance, general execution and scholarship. You may also include additional comments for the author, including concerns about dual publication, research ethics, or publication ethics. If requesting major revision, please articulate the new experiments that are needed.

**Reviewer #1:** (No Response)
Response: Thank you.

**Reviewer #2:** The article it is relavant and interesting, however the reading flow should be improved. The writting of the methodology should also be further improved. I suggest to divide it in clear sections.
Response: Thank you. We have adjusted as advised and put into subheadings; lines 143, 151, 183, 210 and 233
**Reviewer #3:** This paper contains very helpful and interesting data, but the results still lack the quality (in Table 2) that would make them consistent.

Response: Thank you. We have adjusted as advised.
