# Supplementary material for: Ivermectin and doxycycline treatments against Onchocerciasis: Adaptations and impact among semi-nomadic population in Massangam Health District, Cameroon
Source: PLoS Negl Trop Dis. 2023 Jul 12;17(7):e0011463. doi: 10.1371/journal.pntd.0011463 (PMC10365309; doi:10.1371/journal.pntd.0011463)
Supplement: Peer review history

# Peer review: Decision Letter 0

**DOI:** 10.1371/journal.pntd.0011463.r001

**Authors:** Ricardo Toshio Fujiwara, Maria Angeles Gómez-Morales

5 Apr 2023

Dear Dr NDITANCHOU ,

Thank you very much for submitting your manuscript "Test and treat with doxycycline strategy for Onchocerciasis: adaptations and impact among semi-nomadic population in Massangam Health District, Cameroon" for consideration at PLOS Neglected Tropical Diseases. As with all papers reviewed by the journal, your manuscript was reviewed by members of the editorial board and by several independent reviewers. In light of the reviews (below this email), we would like to invite the resubmission of a significantly-revised version that takes into account the reviewers' comments. 

We cannot make any decision about publication until we have seen the revised manuscript and your response to the reviewers' comments. Your revised manuscript is also likely to be sent to reviewers for further evaluation.

When you are ready to resubmit, please upload the following:

[1] A letter containing a detailed list of your responses to the review comments and a description of the changes you have made in the manuscript. Please note while forming your response, if your article is accepted, you may have the opportunity to make the peer review history publicly available. The record will include editor decision letters (with reviews) and your responses to reviewer comments. If eligible, we will contact you to opt in or out.

[2] Two versions of the revised manuscript: one with either highlights or tracked changes denoting where the text has been changed; the other a clean version (uploaded as the manuscript file). 

Important additional instructions are given below your reviewer comments.

Please prepare and submit your revised manuscript within 60 days. If you anticipate any delay, please let us know the expected resubmission date by replying to this email. Please note that revised manuscripts received after the 60-day due date may require evaluation and peer review similar to newly submitted manuscripts.

Thank you again for your submission. We hope that our editorial process has been constructive so far, and we welcome your feedback at any time. Please don't hesitate to contact us if you have any questions or comments.

Sincerely,

Maria Angeles Gómez-Morales, PhD

Academic Editor

PLOS Neglected Tropical Diseases

Ricardo Fujiwara

Section Editor

PLOS Neglected Tropical Diseases

***********************

Reviewer's Responses to Questions

**Key Review Criteria Required for Acceptance?**

As you describe the new analyses required for acceptance, please consider the following:

**Methods**

-Are the objectives of the study clearly articulated with a clear testable hypothesis stated?

-Is the study design appropriate to address the stated objectives?

-Is the population clearly described and appropriate for the hypothesis being tested?

-Is the sample size sufficient to ensure adequate power to address the hypothesis being tested?

-Were correct statistical analysis used to support conclusions?

-Are there concerns about ethical or regulatory requirements being met?

Reviewer #1: The method is well described. The statistical analysis is appropriate. 

Could you please clarify by whom the satellite images were provided?

Could you please give more information regarding the ESRI ArcGIS Explorer live tracking App? Could you please give more details on how it was used? This is not very clear.

Could you please indicate where ivermectin was sourced?

line 212 could you please clarify what statistical test was used to determine the non-normal distribution of intensity data?

Please correct the Error reference at line 119

Could you please add in the supplementary material the individual data concerning the skin snips? I believe this is essential information to be included.

Reviewer #2: - Lines 129-136, the methodology used to identify enchampments should be further detailed. Was it there a systematic approach? How was conducted?

- Line 152-154, How were snips preserved and transported? What were the conditions? Where was the test conducted? 

- Line 156-159, the authors might have missed up ivermectin and doxycycline? Otherwise, I suggest to rephrase that part. 

- Line 185, authors should include in the discussion how this might have affected their results. 

- Line 196-197, what was the denominator (population) considered for that calculation? 

- Lines 205-212, what were the variables considered for the multiple logistic regression?

Reviewer #3: The methods used in this study are well suited to address its key questions. There are no major analyses requested, however (see Results review section) I would suggest adding another table with patient that were present at both beginning and endpoint.

--------------------

**Results**

-Does the analysis presented match the analysis plan?

-Are the results clearly and completely presented?

-Are the figures (Tables, Images) of sufficient quality for clarity?

Reviewer #1: The statistical analysis is sufficiently described.

It would be useful including few additional details in table 1:

1. Although the end-line survey is described in the method section, it would good to indicate that the end-line survey occurred 8 months after the end of treatment

2. Could you please clarify what is Ref?

3. Could you please indicate the percentage of patients who received ivermectin, doxycycline, or no treatment?

Please correct the Error reference at line 212, 227, and 242

At line 207 please use the appropriate symbol for Chi-square and not X2

Reviewer #2: (No Response)

Reviewer #3: The results are clearly presented and understandable for me. I would however suggest that another table be put together in which only the patents are listed that were already present at study onset and to whom the drug intervention (doxycycline or ivm only) was administered. As the authors write, because of the nomadic lifestyle many patients participated only at the endpoint time, so that this obscures the success of the previous treatment.

There are a few minor points that also need attention:

Line 145: sentence incorrect 

Line 178: really all patients could be found again after 8 months? In a semi-nomadic population? Does not match with line 226 (this is plausible)

Line 317: check grammar

Line 330: check spelling: must read “markers”

--------------------

**Conclusions**

-Are the conclusions supported by the data presented?

-Are the limitations of analysis clearly described?

-Do the authors discuss how these data can be helpful to advance our understanding of the topic under study?

-Is public health relevance addressed?

Reviewer #1: line 323-324 the cited reference (10.1002/14651858.CD011146.pub2) doesn't support '[...]evidence on the effectiveness of doxycycline plus ivermectin compared with ivermectin alone in preventing and treating onchocerciasis [...]'. 'Limited evidence of very low quality from two studies indicates that a six‐week course of doxycycline followed by ivermectin may result in more frequent macrofilaricidal and microfilaricidal activity and sterilization of female adult Onchocerca compared with ivermectin alone; however, effects on vision‐related outcomes are uncertain'.

I would be therefore more circumstantial regarding the parasitological effect of doxycycline, especially because the end-line survey occurs only 8 months after treatment. 

If available, it would be very interesting to include information regarding the clinical benefit (itching, presence of microfilaria in the eyes, etc...) or at least mention this point in the discussion.

lines 325-326 I don't think there is enough evidence to support that patients were cured. A longer follow-up would certainly be required to ascertain that a sustained and a long-term absence of microfilariae has indeed taken place. 

lines 329-330 As far as I can see reference 10.1371/journal.pntd.0010380 describes a correlation between skin snip and Ov16 ELISA in children (<10). The article describes such correlation in the context of indicators to stop an MDA campaign based on evaluation in children between 5 to 10 years. Furthermore, the cited article describes a variation of anti-Ov16 IgG4 levels with age and the microfilariae load.

In table table 1 this group of patients is not described, what is the evidence that these testes are correlated in adults? And what is the evidence they can be used as endpoints to assess cure? Please clarify this point.

Reviewer #2: (No Response)

Reviewer #3: iConclusions are appropriate.

--------------------

**Editorial and Data Presentation Modifications?**

Use this section for editorial suggestions as well as relatively minor modifications of existing data that would enhance clarity. If the only modifications needed are minor and/or editorial, you may wish to recommend “Minor Revision” or “Accept”. 

Reviewer #1: (No Response)

Reviewer #2: (No Response)

Reviewer #3: (No Response)

--------------------

**Summary and General Comments**

Use this section to provide overall comments, discuss strengths/weaknesses of the study, novelty, significance, general execution and scholarship. You may also include additional comments for the author, including concerns about dual publication, research ethics, or publication ethics. If requesting major revision, please articulate the new experiments that are needed.

Reviewer #1: I think this work is remarkable. Reaching nomad populations living in remote areas and excluded from the preventive chemotherapy program is certainly a huge challenge.

I am however not entirely convinced by the conclusions and the discussion. Doxycycline is not a first-line treatment for the treatment of onchocerciasis and its parasitological effect is not yet fully proved. Other molecules with an anti-Wolbachia mechanism of action are currently evaluated in clinical trials and there will be more insight regarding the parasitological efficacy in the coming years. It would be therefore important to explain what are the advantages of doxycycline for this population and to justify why in this particular context doxycycline is perceived to be a better option in comparison to ivermectin or moxidectin. Especially, because in this study it is not possible to compare the treatment outcome of patients treated with ivermectin versus doxycycline.

Reviewer #2: This study is very relevant for onchocerciasis elimination and should be published. The rationale of the study is very well written and easy to follow. There are minor comments that I think that should be included in methodology.

Reviewer #3: This is a very important paper, addressing the problems of adherence to yearly mass drug administration of ivermectin for onchocerciasis. It shows that only around 50% of this population had taken part in MDA and therefore analyzed the success of a curative treatment with doxycycline. The data show that in 9/10 patients who received doxycycline, microfilaridermia was absent at the end of the follow up period (endpoint). The data support the use of macrofilaricidal / long-term sterilizing treatments for mobile/hard to reach populations.

--------------------

PLOS authors have the option to publish the peer review history of their article (what does this mean?). If published, this will include your full peer review and any attached files.

If you choose “no”, your identity will remain anonymous but your review may still be made public.

**Do you want your identity to be public for this peer review?** For information about this choice, including consent withdrawal, please see our Privacy Policy.

Reviewer #1: No

Reviewer #2: No

Reviewer #3: Yes: Prof. Achim Hoerauf, Institute for Med. Microbiology, Bonn University Medical Center, Germany

Figure Files:

While revising your submission, please upload your figure files to the Preflight Analysis and Conversion Engine (PACE) digital diagnostic tool, https://pacev2.apexcovantage.com. PACE helps ensure that figures meet PLOS requirements. To use PACE, you must first register as a user. Then, login and navigate to the UPLOAD tab, where you will find detailed instructions on how to use the tool. If you encounter any issues or have any questions when using PACE, please email us at figures@plos.org.

Data Requirements:

Please note that, as a condition of publication, PLOS' data policy requires that you make available all data used to draw the conclusions outlined in your manuscript. Data must be deposited in an appropriate repository, included within the body of the manuscript, or uploaded as supporting information. This includes all numerical values that were used to generate graphs, histograms etc.. For an example see here: http://www.plosbiology.org/article/info%3Adoi%2F10.1371%2Fjournal.pbio.1001908#s5.

Reproducibility:

To enhance the reproducibility of your results, we recommend that you deposit your laboratory protocols in protocols.io, where a protocol can be assigned its own identifier (DOI) such that it can be cited independently in the future. Additionally, PLOS ONE offers an option to publish peer-reviewed clinical study protocols. Read more information on sharing protocols at https://plos.org/protocols?utm_medium=editorial-email&utm_source=authorletters&utm_campaign=protocols

# Author comment: Author response to Decision Letter 0

**DOI:** 10.1371/journal.pntd.0011463.r002

24 Apr 2023

Attachment Submitted filename: Response letter PNTD-D-23-00095.docx Click here for additional data file.

# Peer review: Decision Letter 1

**DOI:** 10.1371/journal.pntd.0011463.r003

**Authors:** Ricardo Toshio Fujiwara, Maria Angeles Gómez-Morales

23 May 2023

Dear Dr. NDITANCHOU ,

Thank you very much for submitting your manuscript "Ivermectin and doxycycline treatments against Onchocerciasis: adaptations and impact among semi-nomadic population in Massangam Health District, Cameroon" for consideration at PLOS Neglected Tropical Diseases. As with all papers reviewed by the journal, your manuscript was reviewed by members of the editorial board and by several independent reviewers. The reviewers appreciated the attention to an important topic. Based on the reviews, we are likely to accept this manuscript for publication, providing that you modify the manuscript according to the review recommendations. 

Please, address all questions and comments raised by the reviewers, some of the questions were raised twice.

Please prepare and submit your revised manuscript within 30 days. If you anticipate any delay, please let us know the expected resubmission date by replying to this email. 

When you are ready to resubmit, please upload the following:

[1] A letter containing a detailed list of your responses to all review comments, and a description of the changes you have made in the manuscript. 

Please note while forming your response, if your article is accepted, you may have the opportunity to make the peer review history publicly available. The record will include editor decision letters (with reviews) and your responses to reviewer comments. If eligible, we will contact you to opt in or out

[2] Two versions of the revised manuscript: one with either highlights or tracked changes denoting where the text has been changed; the other a clean version (uploaded as the manuscript file).

Important additional instructions are given below your reviewer comments. 

Thank you again for your submission to our journal. We hope that our editorial process has been constructive so far, and we welcome your feedback at any time. Please don't hesitate to contact us if you have any questions or comments.

Sincerely,

Maria Angeles Gómez-Morales, PhD

Academic Editor

PLOS Neglected Tropical Diseases

Ricardo Fujiwara

Section Editor

PLOS Neglected Tropical Diseases

***********************

Please, address all questions and comments raised by the reviewers, some of the questions were raised twice.

Reviewer's Responses to Questions

**Key Review Criteria Required for Acceptance?**

As you describe the new analyses required for acceptance, please consider the following:

**Methods**

-Are the objectives of the study clearly articulated with a clear testable hypothesis stated?

-Is the study design appropriate to address the stated objectives?

-Is the population clearly described and appropriate for the hypothesis being tested?

-Is the sample size sufficient to ensure adequate power to address the hypothesis being tested?

-Were correct statistical analysis used to support conclusions?

-Are there concerns about ethical or regulatory requirements being met?

Reviewer #1: The authors have nicely implemented suggestions from reviewers

Reviewer #2: - Ethics, all participants were literate? Otherwise, the approach followed should be detailed here. 

- Community listing in addition to GIS identification of camps is a very approapiate technique using the resources availables. However, in my opinion, the "Procedure" it is not clearly stated. I suggest to clearly divide the different ideas among the different paragraps. It is quite difficult to follow at the moment. 

- Site of analysis or biopsis is not stated. 

- Storage conditions of samples during transportation it is not explained. 

- Microscopy technique used is not stated. 

- Line 248, "Multiple logistic regression", would they mena multiple logistic regressions or amultivariable analysis? This could be clarified. They should also mention if it was an adjusted analysis and the variables they used to adjust for.

Reviewer #3: Ok, as in the original version. I noticed methods have become more detailed, probably at the request of other reviewers.

--------------------

**Results**

-Does the analysis presented match the analysis plan?

-Are the results clearly and completely presented?

-Are the figures (Tables, Images) of sufficient quality for clarity?

Reviewer #1: The authors have nicely implemented suggestions from reviewers

Reviewer #2: - Table 1, the authors should clarify how the OR was calculated and what variables were used. 

- Figure 1 could be substitued with a map of proportion of infectes/proportion of tested/treated per region.

Reviewer #3: A new Table 2 has now been added, this is helpful. However, the numbers do not add up: the numbers in the top line must be the summaries of the lines below, e.g. if Ivermectin “all” =9, of those pos. with microscopy = 0, then those pos. in PCR must have again 9 treated with ivermectin, not 6.

Further, it is written that “All 20 infected individuals completed a 35-day course of doxycycline treatment (table 2). “ One would really like to know how many of them appeared at follow up, and whether they were MF pos. or not, in comparison to the 9 PCR-only positives who received IVM only. Even if numbers are small and do not become significant, it would help interpret the data (btw., I have done a Chi-square out of curiosity if one could obtain a significant difference between doxy treatment and ivermectin-only, and indeed even with these small numbers a difference could become significant if most IVM-treated stayed MF pos. while the doxy (±IVM) treated became MF neg.).

In conclusion, the authors need to rectify Table 2 and tell us about the outcome of the patients which they listed in Table 2.

--------------------

**Conclusions**

-Are the conclusions supported by the data presented?

-Are the limitations of analysis clearly described?

-Do the authors discuss how these data can be helpful to advance our understanding of the topic under study?

-Is public health relevance addressed?

Reviewer #1: The authors have nicely implemented suggestions from reviewers

Reviewer #2: Conclusions are approapriate.

Reviewer #3: Conclusions are fine but need the support as detaled in what I wrote for the Results section.

--------------------

**Editorial and Data Presentation Modifications?**

Use this section for editorial suggestions as well as relatively minor modifications of existing data that would enhance clarity. If the only modifications needed are minor and/or editorial, you may wish to recommend “Minor Revision” or “Accept”. 

Reviewer #1: (No Response)

Reviewer #2: There might be a typo error in line 90: The exclusion of this group from both TTd and ivermectin (ivm MDA treatments represents an obvious equity concern and threatens elimination efforts.

Reviewer #3: (No Response)

--------------------

**Summary and General Comments**

Use this section to provide overall comments, discuss strengths/weaknesses of the study, novelty, significance, general execution and scholarship. You may also include additional comments for the author, including concerns about dual publication, research ethics, or publication ethics. If requesting major revision, please articulate the new experiments that are needed.

Reviewer #1: (No Response)

Reviewer #2: The article it is relavant and interesting, however the reading flow should be improved. The writting of the methodology should also be further improved. I suggest to divide it in clear sections.

Reviewer #3: This paper contains very helpful and interesting data, but the results still lack the quality (in Table 2) that would make them consistent.

--------------------

PLOS authors have the option to publish the peer review history of their article (what does this mean?). If published, this will include your full peer review and any attached files.

If you choose “no”, your identity will remain anonymous but your review may still be made public.

**Do you want your identity to be public for this peer review?** For information about this choice, including consent withdrawal, please see our Privacy Policy.

Reviewer #1: No

Reviewer #2: No

Reviewer #3: No

Figure Files:

While revising your submission, please upload your figure files to the Preflight Analysis and Conversion Engine (PACE) digital diagnostic tool, https://pacev2.apexcovantage.com. PACE helps ensure that figures meet PLOS requirements. To use PACE, you must first register as a user. Then, login and navigate to the UPLOAD tab, where you will find detailed instructions on how to use the tool. If you encounter any issues or have any questions when using PACE, please email us at figures@plos.org.

Data Requirements:

Please note that, as a condition of publication, PLOS' data policy requires that you make available all data used to draw the conclusions outlined in your manuscript. Data must be deposited in an appropriate repository, included within the body of the manuscript, or uploaded as supporting information. This includes all numerical values that were used to generate graphs, histograms etc.. For an example see here: http://www.plosbiology.org/article/info%3Adoi%2F10.1371%2Fjournal.pbio.1001908#s5.

Reproducibility:

To enhance the reproducibility of your results, we recommend that you deposit your laboratory protocols in protocols.io, where a protocol can be assigned its own identifier (DOI) such that it can be cited independently in the future. Additionally, PLOS ONE offers an option to publish peer-reviewed clinical study protocols. Read more information on sharing protocols at https://plos.org/protocols?utm_medium=editorial-email&utm_source=authorletters&utm_campaign=protocols

References

Please review your reference list to ensure that it is complete and correct. If you have cited papers that have been retracted, please include the rationale for doing so in the manuscript text, or remove these references and replace them with relevant current references. Any changes to the reference list should be mentioned in the rebuttal letter that accompanies your revised manuscript. If you need to cite a retracted article, indicate the article's retracted status in the References list and also include a citation and full reference for the retraction notice.

# Author comment: Author response to Decision Letter 1

**DOI:** 10.1371/journal.pntd.0011463.r004

1 Jun 2023

Attachment Submitted filename: Response letter PNTD-D-23-000951R1.docx Click here for additional data file.

# Editor report: Decision Letter 2

**DOI:** 10.1371/journal.pntd.0011463.r005

**Authors:** Ricardo Toshio Fujiwara, Maria Angeles Gómez-Morales

14 Jun 2023

Dear  Dr. Nditanchou,

We are pleased to inform you that your manuscript 'Ivermectin and doxycycline treatments against Onchocerciasis: adaptations and impact among semi-nomadic population in Massangam Health District, Cameroon' has been provisionally accepted for publication in PLOS Neglected Tropical Diseases.

Before your manuscript can be formally accepted you will need to complete some formatting changes, which you will receive in a follow up email. A member of our team will be in touch with a set of requests.

Please note that your manuscript will not be scheduled for publication until you have made the required changes, so a swift response is appreciated.

IMPORTANT: The editorial review process is now complete. PLOS will only permit corrections to spelling, formatting or significant scientific errors from this point onwards. Requests for major changes, or any which affect the scientific understanding of your work, will cause delays to the publication date of your manuscript.

Should you, your institution's press office or the journal office choose to press release your paper, you will automatically be opted out of early publication. We ask that you notify us now if you or your institution is planning to press release the article. All press must be co-ordinated with PLOS.

Thank you again for supporting Open Access publishing; we are looking forward to publishing your work in PLOS Neglected Tropical Diseases.

Best regards,

Maria Angeles Gómez-Morales, PhD

Academic Editor

PLOS Neglected Tropical Diseases

Ricardo Fujiwara

Section Editor

PLOS Neglected Tropical Diseases

***********************************************************

# Editor report: Acceptance letter

**DOI:** 10.1371/journal.pntd.0011463.r006

**Authors:** Ricardo Toshio Fujiwara, Maria Angeles Gómez-Morales

7 Jul 2023

Dear Dr NDITANCHOU,

We are delighted to inform you that your manuscript, "Ivermectin and doxycycline treatments against Onchocerciasis: adaptations and impact among semi-nomadic population in Massangam Health District, Cameroon," has been formally accepted for publication in PLOS Neglected Tropical Diseases.

We have now passed your article onto the PLOS Production Department who will complete the rest of the publication process. All authors will receive a confirmation email upon publication.

The corresponding author will soon be receiving a typeset proof for review, to ensure errors have not been introduced during production. Please review the PDF proof of your manuscript carefully, as this is the last chance to correct any scientific or type-setting errors. Please note that major changes, or those which affect the scientific understanding of the work, will likely cause delays to the publication date of your manuscript. Note: Proofs for Front Matter articles (Editorial, Viewpoint, Symposium, Review, etc...) are generated on a different schedule and may not be made available as quickly.

Soon after your final files are uploaded, the early version of your manuscript will be published online unless you opted out of this process. The date of the early version will be your article's publication date. The final article will be published to the same URL, and all versions of the paper will be accessible to readers.

Thank you again for supporting open-access publishing; we are looking forward to publishing your work in PLOS Neglected Tropical Diseases. 

Best regards,

Shaden Kamhawi

co-Editor-in-Chief

PLOS Neglected Tropical Diseases

Paul Brindley

co-Editor-in-Chief

PLOS Neglected Tropical Diseases
